# Advancing Brain Targeting: Cost-Effective Surface-Modified Nanoparticles for Faster Market Entry

**DOI:** 10.3390/pharmaceutics17050661

**Published:** 2025-05-17

**Authors:** Mariana Gomes, Maria João Ramalho, Joana A. Loureiro, Maria Carmo Pereira

**Affiliations:** 1 LEPABE—Laboratory for Process Engineering, Environment, Biotechnology and Energy, Faculty of Engineering, University of Porto, Rua Dr. Roberto Frias, 4200-465 Porto, Portugal; 2 ALiCE—Associate Laboratory in Chemical Engineering, Faculty of Engineering, University of Porto, Rua Dr. Roberto Frias, 4200-465 Porto, Portugal; 3 Department of Mechanical Engineering, Faculty of Engineering, University of Porto, Rua Dr. Roberto Frias, 4200-465 Porto, Portugal

**Keywords:** blood–brain barrier, drug delivery systems, brain disease treatment, technology transfer

## Abstract

**Background/Objectives**: The blood–brain barrier (BBB) poses a major obstacle to delivering therapeutic agents to the central nervous system (CNS), driving the need for innovative drug delivery strategies. Among these, nanoparticles (NPs) have gained attention due to their ability to enhance drug transport, improve bioavailability, and enable targeted delivery. **Methods**: This paper explores various surface modification strategies employed to optimize NP-mediated drug delivery across the BBB. Specifically, the functionalization of NPs with ligands such as transferrin (Tf), lactoferrin (Lf), protamine, and insulin is discussed, each demonstrating unique mechanisms for enhancing brain-targeting efficiency. In addition, this work provides a comprehensive overview of recent scientific advancements and market strategies aimed at accelerating the adoption of low-cost, surface-modified nanoparticles, ultimately improving patient access to effective CNS treatments. **Conclusions**: Preclinical and in vitro studies have demonstrated the effectiveness of these modifications in increasing drug retention and bioavailability in brain tissues. Additionally, while ligand-conjugated NPs hold significant promise for neuropharmacology, their clinical translation is often hindered by regulatory and economic constraints. Lengthy approval processes can slow market entry, but cost–benefit analyses indicate that surface-modified NPs remain financially viable, particularly as scalable manufacturing techniques and some ligands are cost-efficient.

## 1. Introduction

The treatment of neurological diseases presents significant challenges due to the inherent limitations associated with drug delivery to the brain. One of the primary obstacles is the low permeability of drugs across the BBB, which acts as a selective barrier preventing the passage of most therapeutic agents [1]. This restrictive nature of the BBB severely limits the bioavailability of drugs within the CNS, making it difficult to achieve effective concentrations [2]. Moreover, many neurological drugs suffer from non-specific distribution, leading to high systemic exposure and subsequent adverse side effects [3]. To compensate for these barriers, high doses are often required, which can further amplify toxicity risks and reduce patient compliance [4]. These challenges underscore the urgent need for advanced drug delivery strategies to enhance CNS targeting while minimizing off-target effects [5].

The BBB is a highly specialized structure composed of tightly connected endothelial cells, pericytes, and astrocytes that collectively regulate the passage of substances between the bloodstream and the brain [6]. Its primary function is to protect the brain from harmful toxins, pathogens, and fluctuations in blood composition while ensuring the selective transport of essential nutrients [7]. The tight junctions between endothelial cells prevent the passive diffusion of most hydrophilic molecules, necessitating alternative strategies for drug transport [8]. Several mechanisms facilitate the passage of molecules across the BBB, including passive diffusion for small lipophilic compounds, carrier-mediated transport for essential nutrients, transcytosis, and receptor-mediated transcytosis, which is increasingly exploited for drug delivery [9].

The latter mechanism allows for the exploitation of NPs conjugated with specific ligands on their surface to target endothelial receptors that are overexpressed in the BBB to promote active transport into the brain [10]. Thus, functionalization with targeting ligands, such as peptides, proteins, and antibodies, can enhance receptor-mediated uptake, thereby increasing drug accumulation within the CNS.

Hence, NPs have emerged as a powerful platform for overcoming the limitations of conventional CNS drug delivery methods [11]. Besides allowing for targeted brain delivery, these nanoscale carriers can encapsulate therapeutic agents, protecting them from enzymatic degradation and systemic clearance while facilitating controlled and sustained release. Importantly, NPs can be designed to retain the physicochemical properties of the encapsulated drug, preserving its efficacy while enhancing bioavailability [12].

Although NPs offer significant promise in overcoming these challenges by enhancing drug stability and targeting, several obstacles remain. These include potential immunogenicity, cytotoxicity, scalability issues in production, variability in biodistribution, and the need for extensive regulatory validation [13]. Addressing these challenges is critical for ensuring the successful clinical translation and commercialization of NP-based brain therapies. This review provides an overview of the latest advancements in NP-based drug delivery for brain targeting, presenting a few examples of each ligand, focusing on low-cost surface-modified NPs that hold promise for rapid market entry.

## 2. Nanoparticles as Drug Delivery Systems

NPs are structures typically ranging from 1 to 1000 nm in size, characterized by a high surface-area-to-volume ratio and tunable physicochemical properties. Their small size enables interaction with biological membranes at the cellular and subcellular levels, offering a unique platform for therapeutic and diagnostic purposes [14].

In biomedical applications, NPs provide several advantages, including the protection of therapeutic agents from degradation, controlled and sustained drug release, targeted delivery to specific tissues, and the potential to combine therapeutic and imaging functionalities (theranostics) [15]. They have been explored in cancer therapy, vaccine delivery, gene therapy, regenerative medicine, and the treatment of infectious and neurodegenerative diseases [16].

A wide range of NP formulations have been developed to enhance drug delivery [17]. These formulations are designed to overcome the key challenges of the drugs, such as limited permeability in specific membranes, rapid systemic clearance, and the need for controlled drug release [18]. Each NP type has unique physicochemical properties influencing its interaction with cells and drugs.

Among the most commonly available in the market are liposomes, which are biocompatible phospholipid-based vesicles capable of encapsulating both hydrophilic and lipophilic drugs. Due to their versatility, liposomes have been widely applied in drug delivery, improving bioavailability and reducing the systemic toxicity of several therapeutic agents [19]. Notable examples include Doxil^®^ (doxorubicin liposomal) for cancer treatment, AmBisome^®^ (liposomal amphotericin B) for fungal infections, and DepoDur^®^ (liposomal morphine) for postoperative pain management. These formulations highlight the clinical relevance of liposomes in enhancing drug stability and targeted delivery. Another widely explored category includes polymeric NPs, typically composed of biodegradable materials such as poly(lactic-co-glycolic acid) (PLGA) [20]. PLGA is an FDA-approved synthetic polymer widely used in drug delivery due to its biocompatibility, controlled degradation, and ability to encapsulate both hydrophilic and hydrophobic drugs [21]. These NPs offer controlled drug release and can be further modified by targeting moieties to enhance specificity. Several FDA-approved formulations utilize PLGA-based drug delivery systems, including Lupron Depot^®^ (leuprolide acetate) for prostate cancer and endometriosis, Sandostatin LAR^®^ (octreotide acetate) for acromegaly and neuroendocrine tumors, and Risperdal Consta^®^ (risperidone) for schizophrenia [22].

Metallic NPs, including those made from gold, silver, or iron oxide, have also gained attention due to their potential for drug delivery and imaging applications [23]. These NPs offer unique advantages, such as a high surface-area-to-volume ratios, tunable optical and magnetic properties, and the ability to cross biological barriers, making them ideal for theragnostic applications. Their ability to be functionalized with various ligands enables precise targeting and controlled drug release [24]. Several metallic NP-based formulations have entered clinical trials, with some already being FDA-approved, such as NanoTherm^®^ (superparamagnetic iron oxide NPs - SPIONs) for glioblastoma treatment through magnetic hyperthermia therapy [25]. Additionally, gold NPs are being explored for applications in cancer therapy and photothermal treatments, while silver NPs exhibit antimicrobial and wound-healing properties [26].

Lipid-based carriers, such as solid lipid NPs (SLNs) and nanostructured lipid carriers (NLCs), have also demonstrated promising results for neurodegenerative disease treatment due to their stability and high drug-loading capacity [27]. SLNs and NLCs have been particularly useful in improving the bioavailability of poorly soluble drugs and providing sustained drug release [28]. Commercial formulations such as Doxil^®^ (doxorubicin-loaded LNPs for metastatic ovarian cancer and HIV-associated Kaposi’s sarcoma therapy) demonstrate the real-world applicability of lipid-based NPs in medicine.

### Cost-Effectiveness of Nanoformulations

The cost-effectiveness of NP drug delivery systems is a crucial factor in their widespread adoption in clinical settings. While advanced nanocarriers offer remarkable improvements in drug bioavailability, targeted delivery, and reduced side effects, their commercial viability depends on achieving a balance between production costs and therapeutic benefits. Among the various types, liposomes and polymeric NPs stand out as cost-efficient choices due to their well-established production methods, regulatory approval, and biocompatibility. For example, considering that NP production costs are largely driven by raw material prices and synthesis complexity, PLGA is priced at approximately EUR 60 per gram [29], whereas gold, a key material in metallic NP synthesis, costs around EUR 270 per 500 mg [30], making it more than three times as expensive by weight. These figures exclude downstream processing costs, which are typically more demanding for metallic NPs due to precision synthesis and purification requirements [31]. Moreover, metallic NPs may pose long-term safety concerns due to their non-biodegradable nature and potential for accumulation in organs such as the liver and spleen [32].

Similarly to polymeric NPs, lipid-based NPs (SLNs and NLCs) provide a balance between affordability and performance, particularly in formulations for neurodegenerative diseases, where their ability to enhance brain drug delivery outweighs cost considerations.

## 3. Mechanisms of the Nanoparticles in Brain Targeting

Several factors influence the design of NPs for effective brain targeting. The interplay between size, charge, and surface chemistry directly affects their ability to cross the BBB, interact with target cells, and maintain stability in circulation. Recent studies suggest that optimizing these parameters can significantly enhance drug accumulation in brain tissues while reducing systemic toxicity. Size plays a critical role as NPs typically need to be within the range of 100–200 nm to ensure optimal BBB penetration while avoiding rapid clearance by the immune system [33]. Surface charge is another crucial parameter; a slightly negative or neutral zeta potential is preferred to enhance circulation time, while positively charged NPs may exhibit higher cellular uptake but pose toxicity risks [34]. Biocompatibility is paramount, with materials selected based on their stability, safety profile, and potential for immune activation [35].

**Figure 1:**
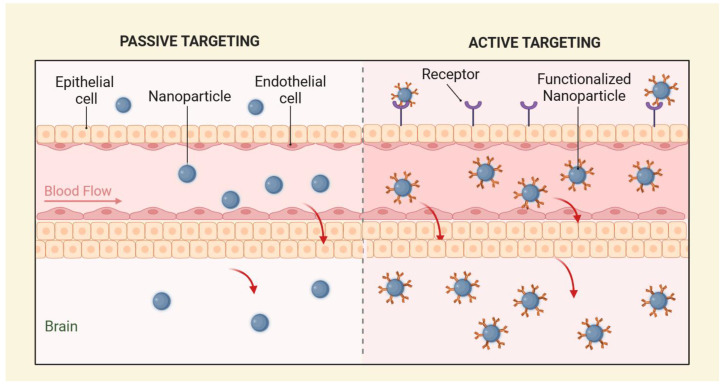
Representation of passive targeting (**left**) and active targeting (**right**).

NPs employ active and passive targeting strategies to enhance drug delivery across the BBB [36], and both targeting strategies are represented in Figure 1. Active targeting leverages molecular interactions between ligands and specific receptors on brain endothelial cells, improving transport efficiency. Conversely, passive targeting relies on a prolonged circulation time and the enhanced permeability and retention (EPR) effect, which primarily occurs in pathological conditions such as tumors, inflammation, or ischemia, where the BBB becomes disrupted. In healthy brain tissue, the intact BBB significantly limits the effectiveness of passive targeting strategies, underscoring the need for alternative active transport mechanisms [17].

Active targeting involves the conjugation of NPs with ligands that specifically interact with receptors on the BBB. One widely studied mechanism is receptor-mediated transport in which NPs functionalized with molecules such as Tf, low-density lipoprotein (LDL) ligands, or insulin-binding ligands interact with specific receptors on endothelial cells, facilitating their transport into the brain [37]. These receptors are often involved in essential processes like nutrient uptake or cellular signaling, making them ideal targets for drug delivery. For example, Tf-functionalized NPs exploit the high expression of transferrin receptors (TfRs) in brain capillaries. Once the NPs bind to the TfRs, they are internalized into the endothelial cells through receptor-mediated endocytosis. This process may involve subsequent transcytosis, where the NP is transported across the endothelial cell and released into the brain tissue, enhancing the delivery of therapeutic agents [38]. LDL receptors and insulin-binding ligands follow a similar mechanism, binding to their respective receptors and facilitating BBB penetration [39].

Another promising active targeting strategy is peptide-mediated transport, which exploits peptides like Angiopep-2 (Ang-2) and RGD. These peptides have a strong affinity for receptors overexpressed on brain capillary endothelial cells (BCECs). Tong et al. (2025) [40] optimized peptide-conjugated LNPs for efficient siRNA delivery across the BBB. Ang-2, which targets LRP-1, was conjugated to a novel LNP formulation (C2), achieving a 2.23% injected dose accumulation in the brain. RGD peptides, on the other hand, target integrins, which are overexpressed in the blood vessels of tumors, making them particularly useful for glioblastoma-targeted drug delivery. Upon binding to these receptors, the peptide-functionalized NPs undergo internalization and subsequent transcytosis, allowing the drug to reach the brain tumor or healthy brain tissue [41].

Passive targeting strategies exploit physiological factors such as prolonged circulation time and leaky vasculature in pathological conditions, such as in inflammatory and tumor tissues [42]. By modifying the physicochemical properties of NPs, researchers aim to extend their half-life in the bloodstream and increase their likelihood of accumulation within brain tissues. One such approach involves coating NPs with polyethylene glycol (PEG), which reduces opsonization and enhances circulation time, thereby improving CNS drug delivery [43]. However, zwitterionic coatings have emerged as an alternative to PEGylation, offering superior stability and enhanced diffusion properties while minimizing undesirable immune interactions [44]. Studies suggest that zwitterion-functionalized NPs exhibit improved permeability across biological barriers compared to their PEG-coated counterparts [45]. Cao et al. (2012) [46] reviewed the advantages of zwitterionic poly(carboxybetaine) (PCB) over non-ionic polymers like PEG for enhancing the performance of NPs in drug delivery applications. The authors highlighted the unique super-hydrophilic nature of PCB, which improves the stability and bioactivity of NPs, while also offering superior permeability across biological barriers such as the BBB.

Additionally, researchers have explored various strategies to enhance NP uptake by brain cells, including using stimuli-responsive systems that trigger drug release in response to specific biological conditions, such as temperature changes, enzymatic activity, or redox potential shifts. These systems offer additional precision in drug delivery, ensuring that therapeutic agents are released only in the desired brain regions, minimizing off-target effects. For instance, pH-sensitive NPs remain stable in circulation but release their cargo upon encountering the slightly acidic microenvironment associated with brain tumors, ensuring localized drug delivery while minimizing systemic exposure [47]. Similarly, enzyme-responsive NPs have been designed to take advantage of elevated protease activity in brain tumors and neurodegenerative diseases, allowing for controlled and tumor-selective drug release [48].

Beyond passive and receptor-mediated transport, focused ultrasound (FUS) combined with microbubble injection has emerged as a non-invasive technique to open the BBB temporarily, facilitating NP penetration into brain tissues [49]. This method has been successfully applied in preclinical models of Alzheimer’s disease (AD) [50] and glioblastoma [51], demonstrating enhanced drug accumulation in previously inaccessible brain regions.

Another innovative approach to enhance NP uptake in the brain is magnetically guided drug delivery, which employs iron oxide-based NPs that respond to externally applied magnetic fields. These NPs can be actively directed to specific brain regions, improving drug accumulation and reducing off-target distribution. Similarly, tumor-treating fields (TTFs)—a technique that uses low-intensity alternating electric fields—have been investigated to enhance NP penetration into glioblastoma cells, improving drug retention and tumor shrinkage in preclinical studies [52].

Emerging biomimetic approaches, such as exosome-coated NPs, provide an innovative strategy for CNS drug delivery. Exosomes—naturally occurring extracellular vesicles—can cross the BBB and deliver therapeutic cargo directly to neurons and glial cells. Studies suggest that exosome-functionalized NPs demonstrate superior BBB penetration and reduced immunogenicity, making them a highly promising RNA and gene therapy platform in neurodegenerative disorders [53].

Beyond targeting, theranostic NPs have emerged as innovative platforms combining imaging and therapeutic capabilities. Such systems enable real-time tracking of NP distribution and treatment monitoring, improving precision and efficacy [54]. For example, superparamagnetic iron oxide nanoparticles (SPIONs) can simultaneously serve as MRI contrast agents and deliver localized hyperthermia therapy, as seen in NanoTherm^®^ applications for glioblastoma [55]. Gold nanoparticles have similarly been investigated for their combined photothermal ablation and imaging capabilities [56]. Incorporating surface modifications can further enhance the targeting efficiency of these theragnostic systems [57].

## 4. Ligands and Surface Modification for Nanoparticles

**Figure 2:**
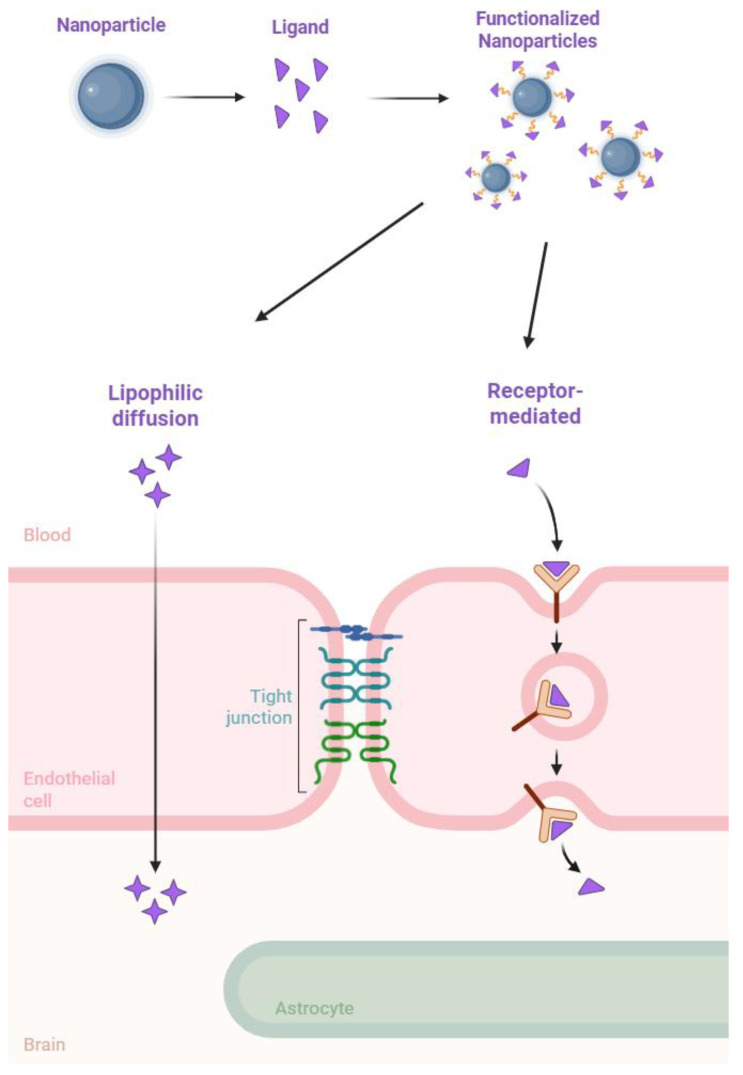
A representation of surface modified nanoparticles’ mechanisms for crossing the BBB.

As mentioned previously, conjugating NPs with various molecules, such as proteins, peptides, and antibodies, is a widely utilized strategy. This approach enables the NPs, along with the encapsulated drug, to cross the BBB and achieve targeted delivery, thereby enhancing the overall efficiency of the treatment (Figure 2).

### 4.1. Surface Modification with Proteins

#### 4.1.1. Transferrin

Tf is a glycoprotein essential for iron transport and has gained significant attention in the field of drug delivery due to its ability to bind to TfRs [58]. These receptors are abundantly expressed on the BBB and certain types of brain tumor cells [59]. By exploiting the receptor-mediated transport mechanism of Tf, researchers have developed innovative NP-based systems capable of crossing the BBB and delivering therapeutic agents directly to the brain. Kumar et al. (2025) [60] developed Tf-modified gemcitabine-loaded PLGA NPs (Tf-GB-PLGA-NPs) for targeted brain cancer therapy, specifically glioma. Surface modification with Tf was designed to exploit the overexpression of TfRs on glioma cells and the BBB, enhancing receptor-mediated endocytosis and BBB permeability. The formulated NPs had an average size of 143 nm, a zeta potential of −25 mV, and a 78% drug entrapment efficiency, ensuring stability and the controlled release of gemcitabine over 24 h. In vitro studies using U87MG glioblastoma cells demonstrated that Tf-GB-PLGA-NPs induced higher apoptosis (61.25%) compared to non-modified NPs (31.61%), confirming superior tumor cell uptake. In vivo pharmacokinetic studies in rats showed that Tf-GB-PLGA-NPs achieved 11.16-fold higher brain bioavailability compared to free gemcitabine and 2.23-fold higher brain bioavailability than non-targeted GB-PLGA-NPs, proving effective BBB penetration. These findings indicate that Tf conjugation significantly improves the therapeutic efficacy of gemcitabine against glioma by enhancing both cellular uptake and brain accumulation.

Ramalho et al. (2023) [61] studied the effect of Tf-conjugated PLGA NPs to deliver temozolomide and bortezomib to glioblastoma cells to promote their efficiency. These resulting NPs have characteristics ideal for GBM cell delivery, including diameters of less than 200 nm (156 ± 3 nm), low polydispersity, and a negative surface charge, as well as controlled and sustained release over 20 days. In vitro studies were performed, and the findings show that GBM cells quickly uptake the NPs.

A study by Neves et al. (2021) [62] aimed to investigate methods of administering curcumin (Cur), a drug recognized for its anti-inflammatory and neuroprotective effects, for treating brain disorders. This study designed a formula of SLNs and NLCs functionalized with Tf to improve and allow penetration through the BBB, resulting in drug delivery to the intended cells. The produced nanoformulations were negatively charged and were less than 200 nm in size, allowing for BBB crossing. The encapsulation efficiency of SLNs was consistently lower than that of NLCs, with the functionalized particles achieving an efficiency of around 70–75%. Stability studies revealed that these particles were stable for up to three months. In vitro experiments on hCMEC/D3 cells revealed that Tf-functionalized Cur-loaded NPs could traverse the BBB more efficiently, with a 1.5-fold increase in permeability compared to non-modified NPs. Moreover, increased entrapment efficiency and cell permeability were observed for the Tf-functionalized NPs compared to the Tf-free NPs. As a result, the developed nanosystems appear to be effective for the brain delivery of Cur, potentially increasing its bioavailability and targeting the brain by adding Tf to the NPs’ surfaces.

In another notable study conducted by Lopalco et al. (2018) [63], Tf-functionalized liposomes were investigated for their ability to enhance dopamine delivery across the BBB, particularly for Parkinson’s disease treatment. These liposomes were developed using a modified dehydration-rehydration method and exhibited a particle size of 180 nm with a positive zeta potential. Also, the NPs showed an encapsulation efficiency of 35%. According to in vitro studies, the functionalized liposomes demonstrated excellent stability and permeability, significantly improving the delivery of dopamine HCL. By leveraging the natural affinity of Tf to its receptors, this study provides a strong foundation for applying Tf-modified liposomes in neurodegenerative disease therapy, where precise drug delivery to the brain is critical.

Another innovative approach in the field of brain drug delivery is the use of dual-targeting NPs. Jian-Qing et al. (2013) [64] performed a study where the potential of liposomes modified with both Tf and folate for glioblastoma treatment was studied. These dual-targeting liposomes encapsulated doxorubicin (DOX), an anticancer agent, and demonstrated efficient BBB penetration and tumor-specific targeting. The NPs had a mean particle size of 180 ± 12.5 nm and were spherical and uniform in size based on an AFM analysis. The drug entrapment efficiency was around 98%, and from the drug release studies, it was possible to observe a sustained release profile. The combination of folate and Tf-targeting mechanisms enabled enhanced drug accumulation in glioma cells while reducing systemic toxicity. In vivo experiments showed a marked reduction in tumor size, improved survival rates, and fewer side effects compared to free DOX. This dual-targeting strategy underscores the potential of combining Tf with other ligands to achieve synergistic effects in drug delivery.

The studies reviewed here underscore the versatility and efficacy of Tf-functionalized NPs in addressing the challenges of CNS drug delivery. By capitalizing on the receptor-mediated endocytosis pathway of Tf, these systems improve drug transport across the BBB and enhance the specificity of drug delivery to target cells within the brain. Incorporating Tf into NP design has demonstrated significant potential in both preclinical and in vitro settings, offering promising avenues for treating CNS disorders and brain tumors.

#### 4.1.2. Lactoferrin

Lf-conjugated NPs have emerged as a viable approach to targeted drug delivery, notably in the treatment of neurodegenerative diseases like Parkinson’s and Alzheimer’s. Several studies have shown that Lf can improve therapeutic drug transport across the BBB due to its ability to bind to specific receptors overexpressed in the brain [65]. The following studies allow for the comprehension of the current advances in the production and development of Lf-conjugated NPs, focusing on their design, effectiveness, and therapeutic promise.

A study developed by Singh et al. (2016) [66] focused on the production of docetaxel (DXT)-loaded SLNs conjugated with Lf for effective brain targeting and brain tumor treatment. The NPs were prepared using an emulsification and solvent evaporation method, and the conjugation of Lf on the SLN surface was performed. The Lf-SLNPs averaged 121 nm in size, and the zeta potential was negative. Cytotoxicity and cellular uptake studies showed that Lf-modified NPs exhibited enhanced apoptotic activity and drug delivery compared to unmodified NPs. In vivo studies were performed in Swiss Albino Mice, and a higher DXT concentration in the brain with Lf-SLNPs was demonstrated compared to SLNs and other nanoformulations in the market. Overall, Lf conjugation provided a key advantage in targeting DXT in the brain, making it a promising drug delivery system.

Xu et al. (2017) [67] developed Lf-coated polysaccharide NPs (Lf-Cur-PSNPs) composed of chitosan hydrochloride, hyaluronic acid (HA), and PEG for targeted glioma therapy. Lf modification was designed to enhance BBB permeability and glioma targeting by binding to Lf receptors (LfR) and LDL receptor-related protein (LRP), which are overexpressed in both BBB endothelial cells and glioma cells. These Lf-functionalized NPs efficiently transported Cur, a potent anticancer agent, to the brain. The Lf-Cur-PSNPs had an average size of 162 nm and a zeta potential of −11.2 mV, indicating stability and a favorable charge for cellular uptake. In vitro studies demonstrated that Lf-Cur-PSNPs exhibited higher uptake in BCECs and C6 glioma cells compared to unmodified PSNPs. Cytotoxicity studies confirmed that Lf-Cur-PSNPs had a stronger inhibitory effect on C6 glioma cells while sparing BCECs. Mechanistic studies revealed that uptake occurred via clathrin-mediated endocytosis, with Lf-dependent internalization in glioma cells. In vivo biodistribution studies in mice showed that Lf-Cur-PSNPs achieved 2.39-fold higher brain accumulation than unmodified PSNPs and remained detectable in the brain for up to 72 h, demonstrating enhanced BBB penetration and prolonged retention. These findings suggest that Lf-modified PSNPs represent a promising dual-targeting nanocarrier for glioma therapy, leveraging Lf and HA-mediated receptor interactions for enhanced brain uptake and glioma specificity.

Li et al. (2018) [68] developed Lf-functionalized PEG-PLGA NPs (Lf-NPs) loaded with shikonin (SHK) for glioma therapy. SHK is a potent anticancer agent, but its poor aqueous solubility and limited BBB permeability restrict its therapeutic potential. Lf modification was designed to enhance BBB transport and glioma cell targeting via receptor-mediated transcytosis through LfR. Lf-NPs were prepared using emulsion solvent evaporation, exhibiting high encapsulation efficiency, sustained drug release, and narrow size distribution. The average size of the Lf-NPs was 174 nm, while the zeta potential was −9.7 mV, indicating good stability and reduced aggregation potential. In vitro studies demonstrated that Lf-NPs showed higher uptake in C6 glioma cells compared to non-modified NPs, indicating receptor-mediated endocytosis. Cytotoxicity assays confirmed that Lf-SHK-NPs induced higher apoptosis rates in glioma cells compared to free SHK due to improved cellular uptake. In vivo biodistribution studies revealed that Lf-SHK-NPs achieved significantly higher brain accumulation, with a 2.8-fold increase in brain concentration compared to non-functionalized NPs. Furthermore, pharmacokinetic analysis indicated that Lf-NPs prolonged SHK circulation time and improved its systemic bioavailability. These findings suggest that Lf-modified PEG-PLGA NPs enhance BBB permeability, prolong drug circulation, and improve glioma targeting, making them a promising platform for brain tumor therapy.

#### 4.1.3. Protamine

Protamine-conjugated NPs have gained attention as a promising tool for improving drug delivery across the BBB [69]. Protamine, a cationic arginine-rich protein, facilitates cellular uptake and enhances the transport of NPs due to its strong electrostatic interaction with negatively charged cell membranes [70]. These properties make protamine-functionalized NPs attractive tools for targeted drug delivery in neurodegenerative diseases, optimizing therapeutic efficacy while reducing systemic toxicity [71].

Dhami et al. (2014) [72] developed protamine-coated PLGA NPs (Pt-PLGA NPs) loaded with cisplatin to enhance BBB penetration and glioblastoma targeting. The Pt-PLGA NPs had an average size of 173.2 ± 7.9 nm and a zeta potential of +10.33 ± 0.03 mV, indicating improved surface charge for BBB penetration compared to non-coated PLGA NPs (140 ± 10.2 nm, −4.69 mV).

In vitro studies demonstrated that Pt-PLGA NPs transported 172.41 ± 15.04 µg of cisplatin across a bovine brain microvessel endothelial cell model, significantly higher than the 110.48 ± 4.71 µg of cisplatin transported by unmodified PLGA NPs and the amount of 20.83 ± 1.65 µg transported by free cisplatin solution, confirming enhanced BBB permeability. In U87 glioblastoma cells, Pt-PLGA NPs exhibited higher cytotoxicity (IC50 = 2.1 µM) compared to PLGA NPs (IC50 = 3.9 µM) and free cisplatin (IC50 = 13.33 µM), indicating superior tumor cell uptake. A cellular uptake analysis confirmed that protamine coating enhanced NP internalization via endocytosis mechanisms. The study concludes that Pt-PLGA NPs significantly improve BBB crossing, drug delivery efficiency, and glioblastoma cytotoxicity, making them a promising tool for brain-targeted chemotherapy.

#### 4.1.4. Insulin

The insulin receptor is highly expressed on brain endothelial cells, allowing insulin-functionalized NPs to engage with this transport pathway and improve drug delivery to the brain [73]. This targeted approach not only enhances CNS drug uptake but also reduces peripheral distribution, increasing therapeutic efficacy for neurological disorders while minimizing systemic side effects.

Ulbrich et al. (2011) [74] examined the use of human serum albumin (HSA) NPs modified with insulin or an anti-insulin receptor monoclonal antibody (mAb) (29B4) to enhance drug transport across the BBB. The study aimed to determine whether these functionalized NPs could facilitate the delivery of loperamide, an opioid that typically does not cross the BBB, by utilizing insulin receptor-mediated transport. The NPs were synthesized, followed by the covalent attachment of insulin or 29B4. Functionalization increased the particle size, with insulin-modified NPs expanding from 153 nm to 190 nm and 29B4-functionalized NPs increasing from 152 nm to 157 nm. The zeta potential remained around −36 mV for both formulations. The in vivo results demonstrated that intravenously administered insulin-functionalized NPs enabled the transport of loperamide across the BBB, producing a noticeable antinociceptive effect, as confirmed by the tail-flick test. A similar outcome was observed when NPs were modified with the 29B4 antibody, further supporting the involvement of insulin receptor-mediated mechanisms in drug delivery to the brain. In contrast, NPs conjugated with a non-specific antibody showed no improvement in loperamide transport, indicating that the targeted approach was necessary for effective drug delivery. Moreover, the pre-administration of free 29B4 antibodies completely blocked NP-mediated transport of loperamide, confirming that the observed effect relied on specific interactions with insulin receptors. These findings indicate that insulin-functionalized NPs can significantly improve the ability of therapeutic agents to cross the BBB, offering an alternative strategy for enhancing drug delivery to the CNS. This approach may be particularly useful in treating neurological disorders requiring efficient brain targeting. However, further investigations are needed to optimize the formulation and evaluate its potential for clinical application.

#### 4.1.5. Apolipoprotein E3

Apolipoprotein E3 (ApoE3)-functionalized NPs represent an advanced approach for drug delivery to the CNS, leveraging the natural ability of ApoE3 to facilitate transport across the BBB [75]. ApoE3 binds to LDL receptor-related protein 1 (LRP1), which is overexpressed in brain endothelial cells [76]. This targeted mechanism enhances drug bioavailability in the CNS, making ApoE3-conjugated NPs a valuable strategy for the treatment of neurodegenerative disorders such as Alzheimer’s and Parkinson’s disease [77].

Athalye et al. (2024) [78] investigated the development of ApoE3-functionalized lipid–drug conjugate NPs (ApoE3@LDC-NP) for enhancing the brain delivery of levetiracetam (LVM), an anti-epileptic drug with limited permeability across the BBB due to its hydrophilic nature. The study aimed to improve LVM bioavailability, brain targeting, and therapeutic efficacy for treating brain tumor-related epilepsy (BTE). The optimized NPs exhibited a 132 nm size and a zeta potential of −16 mV, ensuring stability and efficient cellular uptake. In vitro studies using HEK293 and U87MG cell lines demonstrated no significant cytotoxicity and enhanced uptake in glioblastoma cells, confirming their biocompatibility and brain-targeting potential. Pharmacokinetic and in vivo biodistribution studies revealed that ApoE3@LDC-NP achieved a 2.5-fold increase in brain accumulation compared to free LVM, suggesting effective BBB penetration and a prolonged circulation time. Furthermore, the formulation showed an initial burst release of 20%, followed by sustained drug release for up to 30 h, supporting controlled drug delivery. Compared to non-functionalized LVM formulations, ApoE3-functionalized LDC-NPs demonstrated improved stability, enhanced brain uptake, and sustained drug release, highlighting their potential as an effective brain-targeted therapy for epilepsy management in brain tumor patients. The study concludes that ApoE3@LDC-NP may offer a promising alternative to conventional LVM therapy, though further clinical studies are necessary to evaluate their long-term safety and efficacy.

Topal et al. (2021) [79] developed ApoE-functionalized SLNs (ApoE-SLNs) loaded with donepezil to enhance BBB penetration for AD therapy. Donepezil, an acetylcholinesterase inhibitor, has low BBB permeability and significant peripheral side effects, limiting its therapeutic efficacy. The ApoE functionalization was designed to improve brain targeting by binding to LDL receptors highly expressed on brain endothelial cells and neurons. The ApoE-SLNs had an average size of 134 ± 7 nm and a zeta potential of −18.6 ± 2.1 mV, ensuring colloidal stability and controlled drug release. In vitro studies showed that ApoE-SLNs had significantly higher uptake in primary brain endothelial cells (hCMEC/D3) and SH-SY5Y neuronal cells compared to non-functionalized SLNs. Furthermore, in a co-culture BBB model using rat brain endothelial cells, pericytes, and astrocytes, ApoE-SLNs demonstrated enhanced permeability, confirming receptor-mediated transcytosis. The study concluded that ApoE-functionalized SLNs effectively increased BBB transport of donepezil, offering a potential nanocarrier system for targeted Alzheimer’s therapy with reduced peripheral side effects.

Hartl et al. (2021) [80] developed apolipoprotein E (ApoE)-functionalized polymeric NPs for drug delivery across the BBB using LDL receptor-mediated transcytosis. ApoE, a key ligand in lipid transport, was attached to NPs via two approaches: adsorption using polysorbate 80/poloxamer 188 and covalent bonding. These functionalized NPs effectively transported various drugs, including dalargin, loperamide, DOX, and nerve growth factor, into the brain. The study highlighted that the direct covalent coupling of ApoE to NP surfaces led to significantly higher BBB penetration compared to adsorption-based strategies. The ApoE-functionalized NPs had an average size of 180–220 nm and a zeta potential of −10 to −25 mV, ensuring stability and optimal interaction with BBB receptors. In vitro studies used BBB models composed of brain endothelial cells, astrocytes, and pericytes, demonstrating that ApoE-functionalized NPs had superior transcytosis rates and enhanced cellular uptake compared to non-functionalized NPs. In vivo studies in CNS disease models (Alzheimer’s, Parkinson’s, and cerebral cancer) confirmed higher brain accumulation of ApoE-NPs, leading to improved drug efficacy. These findings suggest that ApoE-modified polymeric NPs are a promising strategy for BBB-targeted drug delivery, leveraging LDL receptor-mediated transcytosis for efficient transport into the brain.

The key ligands and their associated NP types, physicochemical properties, targeting mechanisms, and therapeutic applications are presented in Table 1.

#### 4.1.6. Emerging Protein Ligands

Beyond well-established protein ligands such as Tf, Lf, protamine, and insulin, other proteins and protein-related molecules have been explored to enhance NP targeting and transport across the BBB. Shiga Toxin B (STxB) is the non-toxic B-subunit protein of the Shiga toxin, playing a key role in cell surface recognition and the intracellular transport of the holotoxin [81]. STxB specifically binds to globotriaosylceramide (Gb3) receptors, which are expressed on brain endothelial cells, making it a promising ligand for receptor-mediated transcytosis and facilitating NPs transport into cells. Researchers have investigated STxB-functionalized NPs for CNS drug delivery due to their ability to interact with Gb3 and enhance cellular uptake [82].

These alternative ligands offer promising avenues for improving NP functionalization and expanding the range of targeting strategies for neurological drug delivery [83,84,85].

### 4.2. Surface Modification with Peptides

Peptide-functionalized NPs have emerged as an effective approach to enhancing drug transport across the BBB through receptor-mediated transcytosis. Specific peptides, such as Ang-2, and cell-penetrating peptides (CPPs), can selectively bind to BBB-associated receptors, improving NP uptake and delivery to the CNS. Using these ligands, peptide-conjugated NPs enable more precise targeting of neurological disorders [86,87].

Sepasi et al. (2023) [88] focused on developing GREIRTGRAERWSEKF (CDX)–chitosan NPs to deliver pEGFP-N1 plasmid into the brain parenchyma as a promising approach for treating several neurofunction disorders such as glioblastoma. CDX is a peptide that can identify and bind to the a7 subunit of nicotinic acetylcholine receptor (nAchR), which is highly present in the CNS and spinal cord. The developed CDX-conjugated NPs exhibit a slightly positive zeta potential of 0.9 mV and a size of around 120 nm. In vitro cellular uptake analysis was performed with the rat C6 glioma cell line, and it was demonstrated that the cellular uptake of the nanocomplex depends on the concentration since the presence of the plasmid was intensified with the increase in the concentration of the CDX-nanoformulation. In vivo studies were also performed to evaluate and compare the efficiency of the CDX-modified and non-modified NPs, and it was proven that CDX NPs are capable of crossing BBB and reaching the brain parenchyma, unlike CS NPs. Given the importance of a7nAchR function in diseases like glioma and AD, it is believed that this might be a favorable approach for gene delivery and the treatment of these diseases.

Hoyos-Ceballos et al. (2020) [89] developed PLGA NPs modified with the BBB-penetrating peptide Ang-2 to address the challenge of delivering drugs to the brain. Two strategies were employed to formulate the modified NPs pre- and post-formulation conjugation of Ang-2 to PLGA NPs. The size was 166.4 nm and 177.3 nm, and both represented a negative zeta potential of around -20 mV, which was compatible with systemic administration and BBB penetration. In vivo studies in C57BL/6 mice were conducted to qualitatively evaluate the ability of these NPs to cross the BBB. The results demonstrated that the Ang-2-modified NPs successfully penetrated the BBB and accumulated within various brain regions compared to unmodified NPs, which showed no signal of relevant accumulation of NPs. These results suggested that the conjugation of NPs with Ang-2 increases the targeted delivery of the NPs to the brain and, consequently, is a promising treatment for brain diseases.

Another interesting study developed by Kuo et al. (2019) [90] optimized the production of liposomes modified with the transactivator of transcription (TAT) peptide and loaded with anti-apoptotic drugs to target hippocampal neurons and prevent tau-hyperphosphorylated neurodegeneration. The optimized TAT-modified NPs had an average size of 158.5 nm and were negatively charged (−27.5 mV). Adding TAT peptides to drug-loaded liposomes greatly increased their capacity to pass the BBB. The in vivo study demonstrated that TAT-CL/PA-lip achieved higher BBB penetration and brain tissue distribution than liposomes without TAT peptide, highlighting the TAT peptide’s role in enhancing diffusion and carrier-mediated transport, reducing the required drug dose for therapeutic efficacy. The TAT modification amplified the liposome’s anti-apoptotic, anti-inflammatory, and neuronal growth-supporting effects, making it a promising drug delivery system for combating neurotoxicity and tau-related neurodegeneration in AD.

Fan et al. (2018) [91] developed a novel system of PLGA-PEG NPs modified with B6 peptide (CGHKAKGPRK) to target the TfR and loaded it with Cur (PLGA-PEG-B6/Cur) to address Cur’s low bioavailability and limited ability to cross the BBB. The study’s primary goal was to enhance Cur’s therapeutic efficacy for AD by improving its delivery to the brain. The optimized NPs were characterized by a size of under 150 nm, a relatively high drug-loading capacity of 15.6%, and a biphasic release profile, releasing 30% of the drug within the first hour and sustaining release up to 78% over 72 h. In vitro studies demonstrated that the PLGA-PEG-B6/Cur NPs achieved significantly greater cellular uptake than non-modified NPs and native Cur, with uptake increasing in dose- and time-dependent manners. In vivo tests on APP/PS1 transgenic mice further validated their efficacy, showing reduced amyloid-beta (Aβ) deposition and tau hyperphosphorylation in the hippocampus alongside improved spatial learning and memory in Morris water maze experiments. Compared to unmodified NPs and native Cur, the B6-modified system showed superior results. These findings highlight the potential of PLGA-PEG-B6/Cur NPs as a promising tool for advancing AD treatment.

Huang et al. (2017) [92] developed PLGA NPs loaded with an Aβ generation inhibitor (S1 peptide) and Cur, further modified with a cyclic CRTIGPSVC (CRT) peptide to improve BBB penetration and enhance therapeutic efficacy for AD. The CRT-modified NPs (CRT-NP-S1 + Cur) had an average size of 140 nm and a zeta potential of −26 mV. In vitro studies demonstrated significantly enhanced cellular uptake of CRT-conjugated NPs compared to non-modified ones. In vivo studies in mice with transgenic Alzheimer’s showed that CRT-NP-S1 + Cur NPs exhibited superior performance, reducing Aβ burden, reactive oxygen species (ROS), and inflammatory cytokines (TNF-α and IL-6) while increasing synapse density and improving cognitive functions in memory tests. Compared to non-modified NPs, CRT functionalization significantly improved brain targeting and therapeutic outcomes. These findings highlight the potential of using CRT-modified NPs as an effective strategy for enhancing the delivery of therapeutic agents across the BBB for Alzheimer’s treatment.

Barbara et al. (2017) [93] developed PLGA NPs loaded with Cur and performed surface modification with the g7 glycopeptide to enhance BBB penetration and improve their efficacy against AD. The optimized g7-NPs-Cur had an average size of 200–250 nm with a zeta potential of approximately −13 mV, indicating successful surface modification and a stable formulation. In vitro studies showed that g7-NPs-Cur exhibited no cytotoxic effects and significantly reduced Aβ aggregation while promoting Aβ disaggregation compared to non-modified NPs and free Cur. Additionally, g7-NPs-Cur provided enhanced protection against oxidative stress and inflammation, demonstrating superior efficacy over the unmodified formulations in reducing Aβ-related pathology and restoring neuronal health. These findings suggest that g7-functionalized NPs represent a promising strategy for delivering Cur to the brain and mitigating AD pathology.

Li et al. (2018) [94] developed a dual-functionalized NP system, cFd-Lip/PTX, designed to improve paclitaxel (PTX) delivery for glioma treatment. These NPs, modified with both the dNP2 peptide and pH-sensitive folic acid (FA), exhibited a size of approximately 104 nm and a zeta potential of −6 mV at a neutral pH, which shifted to +6.44 mV under acidic conditions due to FA cleavage. This dual-modification strategy initially enabled FA to aid in tumor targeting while the dNP2 peptide enhanced BBB permeability and facilitated deeper drug penetration into glioma cells. Compared to single-ligand-modified or non-cleavable NPs, the cFd-Lip/PTX formulation demonstrated superior cellular uptake and cytotoxicity under the acidic conditions of the tumor microenvironment. In vivo studies further confirmed that cFd-Lip/PTX achieved greater drug accumulation in glioma tissues and improved therapeutic efficacy compared to other formulations. These findings highlight the potential of cFd-Lip/PTX to overcome the dual challenges of BBB transport and tumor targeting in glioma therapy.

Some other peptides were also studied. Rabies virus glycoprotein (RVG) is a neurotropic peptide known for its high affinity to the nAChR on neuronal cells, making it a promising ligand for facilitating BBB penetration [95]. Penetratin peptide, derived from the Antennapedia homeodomain, is a well-established CPP that enhances translocation across cellular membranes, thereby improving the intracellular delivery of therapeutic agents [96,97]. Nerve growth factor (NGF), a neurotrophin that binds to TrkA receptors, plays a critical role in neuronal survival and regeneration, making it an ideal ligand for neurodegenerative disease therapies [98]. Similarly, cationic peptides, due to their strong electrostatic interactions with negatively charged cell membranes, promote enhanced cellular uptake and have been utilized to improve drug transport across the BBB [99].

Lastly, hexapeptides have gained attention for their ability to enhance receptor-mediated targeting, particularly in peptide-based drug delivery systems where precise cellular interaction is required [100]. These ligands, when conjugated to NPs, significantly enhance targeting specificity, improve drug bioavailability, and reduce off-target effects, making them valuable tools for precision medicine in both neurological treatments. Table 2 describes different peptide ligands and their respective applications.

### 4.3. Surface Modification with Antibodies

Antibody-functionalized NPs provide a highly specific method for overcoming the challenges of BBB permeability, offering a targeted approach to brain drug delivery [101]. By conjugating mAb or antibody fragments to NPs, these systems can engage with specific receptors, such as Tf or insulin receptors [102]. This high degree of specificity improves drug localization within the brain, enhances therapeutic effectiveness, and reduces adverse systemic effects, making antibody-conjugated NPs a powerful tool for neurological disease treatment.

Gandhi et al. (2019) [103] focused on designing IGF-II-functionalized cationic liposomes to improve the delivery of the p11 gene to the brain to overcome the challenges associated with crossing the BBB. The liposomes were prepared with arginine- or histidine-modified lipids to enhance intracellular delivery by promoting endosomal escape. IGF-II mAb were attached to the surface of the liposomes to enable specific interaction with IGF-II receptors on the BBB. The optimized IGF-II-modified liposomes had sizes of 137.9 nm for arginine formulations and 140.6 nm for histidine formulations, with zeta potentials of 21.8 mV and 14.9 mV, respectively. Brain distribution studies demonstrated that IGF-II-functionalized liposomes showed significantly higher accumulation in brain tissues than non-functionalized liposomes, confirming improved BBB transport. A Western blot analysis further revealed that liposomes modified with arginine and IGF-II produced the highest expression of p11 in the hippocampus, outperforming histidine-modified liposomes and naked DNA. This study highlights the potential of IGF-II-modified liposomes for targeted gene delivery to the brain.

Kim et al. (2018) [104] presented a strategy to improve the brain delivery of nucleic acids by designing TfR-targeted cationic liposomes, referred to as scL nano complexes. These nanocomplexes were modified with a single-chain variable fragment (scFv) antibody, enabling targeted interaction with TfR on BBB endothelial cells. The scL nano complexes were optimized to achieve a particle size of around 100 nm and a positive zeta potential, ensuring the effective encapsulation of nucleic acids and enhanced cellular uptake. In in vitro studies, scL nano complexes demonstrated significantly higher uptake in cells than untargeted liposomes, confirming the functional impact of TfR targeting. Complementary in vivo experiments revealed that scL nano complexes accumulated more extensively in brain tissues, with strong fluorescence signals observed in regions such as the hippocampus and cortex. When used to deliver anti-TNF-alpha interfering RNA (siRNA), these nano complexes effectively reduced neuroinflammation and neuronal apoptosis in a mouse lipopolysaccharide (LPS)-induced endotoxemia model. The treatment also improved survival rates relative to animals treated with non-targeted formulations. This work highlights the crucial role of TfR-targeted surface modification in achieving the efficient delivery of therapeutic agents to the brain.

Loureiro et al. (2017) [105] developed SLNs functionalized with the anti-Tf receptor OX26 mAb and the LB 509 mAb for targeted brain delivery and, consequently, for the treatment of AD. Functionalization was carried out with the aim of enhancing the ability of the NPs to cross the BBB and deliver therapeutic agents such as resveratrol, grape seed, and grape skin. The characteristics of the SLNs varied depending on the conjugation. The LB 509 mAb-conjugated SLNs showed a 249 ± 1 nm size and a zeta potential of −5.0 ± 0.1 mV. In comparison, the OX26 mAb-conjugated SLNs exhibited a larger size of 254 ± 17 nm and a zeta potential of −4.0 ± 0.1 mV, reflecting differences in surface modification. In vitro studies using a human brain-like endothelial cell model demonstrated that OX26-functionalized SLNs exhibited the highest cellular uptake and transcytosis efficiency compared to LB 509-functionalized and non-functionalized SLNs. These results highlight the potential of mAb-functionalized SLNs, particularly those conjugated with OX26 mAb, for targeted drug delivery to the brain.

Loureiro et al. (2016) [106] designed PLGA NPs functionalized with two mAb, OX26 and DE2B4, to target TfR and Aβ aggregates for the treatment of AD. The optimized NPs without antibody functionalization had a size of 153 ± 2 nm and a zeta potential of −10.1 ± 0.4 mV. Functionalization with OX26 mAb increased the size to 163 ± 3 nm, while dual functionalization with OX26 and DE2B4 mAbs further increased the size to 166 ± 2 nm, and the zeta potential remained almost stable (−13 ± 1 mV). In vitro studies using porcine brain capillary endothelial cells (PBCECs) demonstrated that dual-modified NPs showed superior cellular uptake compared to NPs with single antibody conjugation or without antibody functionalization. Including both antibodies increased transport efficiency through receptor-mediated transcytosis and specific binding to Aβ aggregates. These findings highlight the potential of dual-functionalized PLGA NPs for improving BBB permeability and targeting Aβ pathology in AD. Table 3 describes different antibody-functionalized NPs and their respective applications.

Other antibodies have also been explored for NP functionalization, including anti-LRP1 [107], anti-Aβ [108], anti-CD133 [109], anti-AChR [110], anti-MCT [111], anti-neuropilin-1 (NRP1) [112], anti-CD98 [113], and anti-VEGF [114], further expanding the range of targeted approaches for BBB permeability enhancement and brain drug delivery.

Although EGFR is not a classical BBB transporter, it has been explored as a potential target in neurodegenerative diseases due to its role in neuroinflammation and cell signaling. In Alzheimer’s disease, EGFR expression may be upregulated in reactive astrocytes and inflamed brain regions. A few studies have investigated anti-EGFR antibody-functionalized NPs to modulate EGFR-related pathways or improve brain-specific delivery under pathological conditions [115].

### 4.4. Surface Modification with Other Ligands

In addition to proteins, peptides, and antibodies, various other ligands can be utilized to functionalize NPs for enhanced brain delivery. Small molecules, such as folate [85] and mannose [116], play a crucial role in targeting specific receptors overexpressed on brain endothelial cells, facilitating receptor-mediated transcytosis across the BBB. Furthermore, vitamins like vitamin B12 (cobalamin) have been explored for brain targeting due to their natural ability to cross the BBB via receptor-mediated endocytosis, making them valuable for NP functionalization [117]. Nucleic acids, particularly aptamers and small siRNA, offer high binding specificity, allowing for the precise targeting of brain cells and therapeutic gene silencing [118].

### 4.5. Surface Modification with Chitosan

Chitosan-functionalized NPs are a promising platform for brain-targeted drug delivery, utilizing chitosan’s biocompatibility, biodegradability, and mucoadhesive properties to enhance BBB permeability [119]. Chitosan has been shown to transiently open tight junctions in the BBB, facilitating increased drug absorption into the CNS. Additionally, its ability to interact with endothelial cells and prolong the NP retention time further supports its role as an effective ligand for improving the bioavailability of therapeutic agents in neurodegenerative disease treatments [120].

Wang et al. (2016) [121] developed chitosan-functionalized pluronic micelles (MYR-MCs) loaded with myricetin (MYR) to improve BBB penetration and glioblastoma therapy. The chitosan modification increased the positive charge density, enhancing cellular uptake and aqueous dispersibility. These MYR-MCs had an average size of 51.5 ± 13.2 nm and a zeta potential of 22.38 ± 4.15 mV, indicating good stability and surface charge properties favorable for BBB transport. In vitro studies demonstrated that MYR-MCs significantly increased cellular uptake in Caco-2 cells (3.27-fold higher than free MYR) and exhibited enhanced cytotoxicity against DBTRG-05MG glioblastoma cells. The IC_50_ value for MYR-MCs was 17.26 µM, much lower than that of free MYR (36.65 µM), confirming improved anticancer potency. In vivo studies in athymic nu/nu mice bearing DBTRG-05MG glioblastoma tumors showed that MYR-MCs inhibited tumor growth by 33.5% at a dose of 25 mg/kg after 14 days, significantly outperforming free MYR. Brain uptake studies revealed that MYR-MCs achieved twice the brain concentration compared to free MYR after oral administration, demonstrating improved BBB penetration. Histopathological analysis confirmed that MYR-MCs increased apoptosis and necrosis in glioblastoma tissue while upregulating pro-apoptotic proteins BAX and BAD and downregulating anti-apoptotic Bcl-2. Furthermore, toxicity assessments showed no significant damage to the liver, heart, or kidneys. These findings suggest that chitosan-functionalized pluronic micelles offer an effective and safe nanocarrier system for glioblastoma therapy, significantly improving BBB transport and tumor targeting.

Ramalingam et al. (2015) [122] designed a novel delivery system using SLNs coated with N-trimethyl chitosan (TMC) to enhance the oral bioavailability and brain uptake of Cur. The optimized TMC-SLNs were characterized by a 412.0 ± 79.7 nm particle size and a positive zeta potential of +35.7 ± 1.03 mV. Compared to non-coated SLNs, these TMC-coated NPs exhibited a controlled release profile, preventing the rapid release of Cur in acidic environments while ensuring sustained release under intestinal conditions. In vivo studies in mice showed that Cur-loaded TMC-SLNs significantly improved Cur’s oral bioavailability (23.07%) and resulted in higher concentrations in brain tissue, particularly in the brain parenchyma, when compared to both unmodified SLNs and free Cur. These findings highlight the potential of TMC-SLNs as an effective platform for enhancing curcumin delivery to the brain and overcoming its limitations in oral absorption.

Mahanta et al. (2024) [116] designed and tested mannose-functionalized chitosan-coated PLGA NPs (CHTMAN-PLGA-CBD) to enable the targeted delivery of cannabidiol (CBD) and brain-derived neurotrophic factor (BDNF) to the brain for treating AD. The optimized NPs had an average size of 306 ± 8.12 nm and a zeta potential of +31.7 ± 1.53 mV, demonstrating the effectiveness of the surface coating with mannose-functionalized chitosan. These particles showed a sustained release of CBD over 22 days, with a cumulative release of 91.68%, while encapsulating the BDNF plasmid (pBDNF) with an efficiency of 74.35 ± 5.34%. In vitro studies revealed that these mannose-coated NPs achieved significantly higher cellular uptake and BDNF expression than non-modified NPs, with a fourfold increase in transfection efficiency in brain endothelial cells, astrocytes, and neurons. The mannose coating enhanced GLUT-1 receptor-mediated targeting, improving brain-specific delivery. These results suggest that CHTMAN-PLGA-CBD NPs offer a promising strategy for targeting the brain and addressing the challenges of Alzheimer’s treatment.

## 5. Market Implications

Lengthy regulatory processes and clinical validation requirements often hinder the market entry of brain-targeted NP-based therapies. However, various strategies can accelerate their approval and commercialization, particularly for low-cost, surface-modified NPs. Regulatory agencies such as the FDA and the European Medicines Agency (EMA) have established expedited approval pathways to facilitate the development of innovative treatments for neurological diseases [123]. These mechanisms are particularly beneficial for low-cost, surface-modified NP-based drug delivery systems targeting brain diseases as they offer superior efficacy while maintaining affordability. By leveraging these pathways, companies can reduce time to market and bring much-needed, cost-effective CNS therapies to patients more rapidly [124]. In parallel, it is essential to recognize that nanomaterials, due to their distinct physicochemical properties, demand more rigorous safety and quality assessments than conventional drug formulations. Factors such as nanoscale size, increased surface reactivity, and potential for unexpected biodistribution may result in immunogenicity, cytotoxicity, or off-target accumulation. These biological impacts necessitate extensive toxicological evaluations, long-term safety monitoring, and batch-to-batch reproducibility validation. Regulatory guidelines require a detailed characterization of parameters such as particle size distribution, zeta potential, surface ligand integrity, and aggregation behavior [33]. Furthermore, NP interactions with the immune system and biological membranes must be studied under both physiological and pathological conditions. It is even more relevant when the NPs are surface-modified with specific ligands, allowing their accumulation in specific tissues. Integrating BBB models into in vitro/in vivo correlation studies and predictive toxicology tools early in development can help reduce risks and streamline approval timelines.

The production of NPs involves multiple steps, including synthesis, functionalization, purification, and large-scale manufacturing, all of which must be optimized for cost efficiency. The scalability of NP manufacturing has improved with advancements in microfluidics, continuous flow synthesis, and high-throughput production techniques, which reduce variability and ensure batch-to-batch consistency while keeping production costs low. However, the economic feasibility of each NP type varies depending on raw material costs, functionalization complexity, and regulatory compliance requirements [125].

For instance, liposomes and polymeric NPs modified with cost-effective ligands are relatively affordable, while metallic and magnetic NPs requiring complex modifications tend to have higher production costs due to material expenses and additional functionalization needs [126]. Surface modifications such as chitosan, Lf, Tf, and other low-cost ligands can significantly improve targeting specificity without adding substantial costs to the production process. Compared to traditional drug delivery systems, NP-based approaches provide higher bioavailability, prolonged circulation time, and reduced frequency of administration, all of which contribute to a more cost-effective treatment regimen [126].

By focusing on affordable and scalable surface modification techniques, the financial burden on both healthcare providers and patients can be minimized, making these advanced therapies more effective and more widely accessible while maintaining commercial viability. Table 4 outlines the estimated production costs for different low-cost surface-modified NP types. It was estimated the surface modification with 100 ligands per NP, and a solution containing 1 mg/mL of PLGA NPs, using a PLGA with a 24 kDa.

Overall, the table demonstrates that protein-based ligands such as protamine, Tf, and Lf are generally more economical compared to mAb primarily due to simpler production processes, higher availability, and lower purification costs associated with proteins versus the complex manufacturing and validation requirements of antibodies.

To ensure commercial viability, large-scale production must integrate automation, in-line quality control, and the use of cost-effective raw materials, all while adhering to Good Manufacturing Practice (GMP) standards. Additionally, leveraging readily available, low-cost surface modification agents such as natural ligands, peptides, and small molecules can significantly reduce production costs while maintaining therapeutic efficacy. Addressing these technical and regulatory challenges is essential for the successful market adoption of low-cost surface-modified NPs, ultimately enabling affordable, high-impact treatments for neurological disorders with faster regulatory approval [142].

Despite the significant progress in developing ligand-functionalized NPs for CNS drug delivery, several challenges continue to hinder their clinical translation. One major barrier is reproducibility, particularly in maintaining consistent ligand density, orientation, and bioactivity across production batches. Small deviations can lead to large differences in biodistribution or therapeutic outcomes, complicating regulatory approval [143]. Scalability also presents a critical challenge, as many ligand-conjugation strategies developed in academic settings rely on complex chemistries or purification techniques that are not easily transferable to industrial manufacturing [144]. Ensuring batch-to-batch consistency while maintaining ligand functionality at scale remains a key hurdle.

Patient variability, including differences in receptor expression, metabolic rates, and immune responses, further complicates translation. Ligands effective in preclinical animal models may not exhibit the same targeting efficiency in diverse human populations, especially when dealing with heterogeneous diseases like Alzheimer’s [145]. Addressing these challenges will require not only advances in formulation science but also the development of standardized protocols, scalable manufacturing platforms, and robust preclinical models that better mimic human physiology.

## 6. Challenges, Opportunities, and Future Directions

The development of surface-modified NPs for brain-targeted drug delivery presents a complex landscape of challenges and opportunities that influence their clinical translation and market adoption.

The market for nanomedicine is experiencing rapid growth. According to Fortune Business Insights, the global nanomedicine market was valued at USD 241.82 billion in 2024 and is projected to reach USD 570.98 billion by 2032, exhibiting a compound annual growth rate (CAGR) of 11.7% [146]. Similarly, Precedence Research estimates that the market size will grow from USD 209.43 billion in 2024 to approximately USD 627.03 billion by 2034, reflecting a CAGR of 11.59% [147].

The NP-based drug delivery sector is also benefiting from increasing demand for personalized medicine, minimally invasive therapies, and neurodegenerative disease treatments. Despite this promising growth, the competitive landscape is becoming increasingly crowded, necessitating continuous innovation and differentiation for new entrants [148].

Demonstrating the clinical efficacy and safety of NP-based therapies remains challenging. The translation from laboratory successes to clinical applications often encounters hurdles due to the complex interactions of NPs within biological systems. Regulatory bodies, such as the FDA, have stringent requirements for nanomedicines, necessitating comprehensive preclinical studies and multi-phase clinical trials. Designing and executing robust clinical trials that adequately address the unique properties of NPs require significant time and resources. Collectively, these factors can result in extended development phases, with time-to-market estimates often exceeding those of traditional pharmaceuticals [149]. The lack of standardized evaluation protocols for NPs also further complicates the approval process, potentially delaying market entry [150].

The pathway to commercialize NP-based drug delivery systems is often prolonged. Factors contributing to extended timelines include regulatory scrutiny, manufacturing scalability, and clinical trial complexity. Transitioning NP production from the laboratory scale to the industrial scale involves overcoming challenges related to reproducibility, quality control, and cost-effectiveness.

In conclusion, while surface-modified NPs hold significant promise for brain-targeted drug delivery, addressing patenting challenges, navigating a competitive market, ensuring clinical validation, and managing extended time to market are critical factors that need to be strategically managed to fully realize their potential.

## 7. Conclusions

Low-cost, surface-modified NPs represent a transformative approach to addressing the long-standing challenges associated with drug delivery to the brain. Their ability to cross the BBB via targeted mechanisms has revolutionized the treatment landscape for CNS disorders [151]. By employing surface modifications with affordable and biocompatible ligands—such as Tf, Lf, protamine, and chitosan—researchers have developed highly efficient, scalable NP systems capable of enhancing drug bioavailability, minimizing systemic toxicity, and improving therapeutic efficacy [152].

The integration of such NPs into neurotherapeutics not only offers scientific and clinical advancements but also aligns with the urgent need for cost-effective healthcare solutions [153]. Scalability in production, simplified synthesis methods, and the use of economical surface ligands ensure that these technologies are accessible to both developed and resource-limited healthcare settings. According to cost comparisons, ligands like protamine, chitosan, and insulin are among the most affordable in contrast to expensive mAb and specialized peptides such as OX26 or Ang-2. This pricing advantage suggests that NPs functionalized with low-cost protein ligands may reach the market more rapidly, offering a pragmatic route to clinical application without compromising therapeutic efficacy.

This paradigm shift is particularly crucial in the context of global health equity, where economic feasibility can determine the reach and impact of advanced therapies.

By leveraging these advanced targeting strategies, NPs hold immense potential for transforming the treatment of neurological diseases. These advancements address the challenges associated with BBB permeability, offering higher specificity, enhanced bioavailability, and reduced systemic toxicity. With ongoing research and clinical validation, these innovative NP systems may pave the way for more effective therapies in neurodegenerative diseases, brain tumors, and CNS infections, significantly improving patient outcomes [154].

As the field progresses, continued interdisciplinary collaboration and investment in regulatory and market strategies will be critical. Through targeted innovation and a focus on affordability, surface-modified NPs are poised to deliver scalable, high-impact treatments that could redefine the future of brain-targeted drug delivery worldwide.

Finally, NPs targeting the brain present a highly promising, cost-effective strategy to overcome the long-standing challenges of central nervous system drug delivery. By utilizing affordable and biocompatible ligands, these NPs enhance BBB permeability and drug specificity without substantially increasing production costs. This balance between efficacy and economic feasibility can accelerate clinical translation, especially when combined with scalable manufacturing methods and regulatory incentives for innovative neurological therapies. Therefore, we believe that prioritizing low-cost surface modifications in NP design offers a pragmatic and impactful approach to improving accessibility to effective treatments for neurological diseases.

## Figures and Tables

**Table 1:** Overview of protein-functionalized NPs for brain-targeted drug delivery, highlighting NP types, size, zeta potential, targeting mechanisms, and therapeutic applications.

Ligand	Type of NP	Size (nm)	Zeta Potential	Function /Target	Application	Ref.
Tf	PLGA NPs	143	Negative	targets TfR for receptor-mediated endocytosis	glioblastoma therapy	[60]
	PLGA NPs	156	Negative	improves BBB permeability	glioblastoma therapy	[61]
	SLNs and NLCs	<200	Negative	improves BBB permeability	inflammatory and neuroprotective effects	[62]
	liposomes	180	Positive	improves BBB permeability	Parkinson’s disease treatment	[63]
	liposomes	180	NA	improves BBB penetration and tumor-specific targeting	brain tumor treatment	[64]
Lf	SLNs	121	Negative	improves BBB penetration and tumor-specific targeting	brain tumor treatment	[66]
	Polysaccharide NPs	162	Negative	enhances BBB permeability and glioma targeting	brain tumor therapy	[67]
	PEG-PLGA NPs	174	Negative	enhances BBB permeability and glioma targeting	brain tumor therapy	[68]
Protamine	PLGA NPs	173.2	Positive	facilitates AMT for enhanced BBB permeability	brain tumor therapy	[72]
Insulin	HSA NPs	157–190	Negative	improves BBB penetration	neurological disorder treatment, CNS drug delivery enhancement	[74]
ApoE3	Lipid-Drug Conjugate NPs	132	Negative	facilitates transport across the BBB	brain tumor-related epilepsy	[78]
	SLNs	134	Negative	enhances BBB penetration	Alzheimer’s disease treatment	[79]
	Polymeric NPs	180–220	Negative	enhances BBB penetration	neurological disorder treatment	[80]

**Table 2:** Overview of peptide-functionalized NPs for brain-targeted drug delivery, highlighting NP types, size, zeta potential, targeting mechanisms, and therapeutic applications.

Ligand	Type of NP	Size (nm)	Zeta Potential	Function/Target	Application	Ref.
Ang-2	PLGA NPs	166.4–177.3	Negative	Enhances BBB penetration and improves drug delivery to brain	Brain drug delivery; treatment of neurological disorders	[88]
CDX	Chitosan NPs	120	Positive	Binds to α7 subunit of nAChR, facilitating BBB crossing	Gene delivery for glioblastoma and neurodegenerative diseases	[87]
TAT (Transactivator of Transcription)	Liposomes	158.5	Negative	Enhances BBB penetration and brain tissue distribution	Anti-apoptotic drugs; AD treatment	[89]
B6 Peptide	PLGA-PEG NPs	<150	Not specified	Improves Cur delivery and increases brain uptake	Alzheimer’s treatment; Cur delivery	[90]
CRT	PLGA NPs	140	Negative	Improves BBB penetration and enhances Alzheimer’s therapy	Alzheimer’s treatment; Aβ reduction	[91]
g7 Glycopeptide	PLGA NPs	200–250	Negative	Enhances BBB penetration and reduces Aβ aggregation	Alzheimer’s treatment; oxidative stress reduction	[92]
dNP2 Peptide	Liposomes	104	Negative/Positive (pH-Sensitive)	Allows for dual-functional modification for glioma therapy, enhances BBB penetration	Glioma therapy; targeted PTX delivery	[93]

**Table 3:** Overview of antibody-functionalized NPs for brain-targeted drug delivery, highlighting NP types, size, zeta potential, targeting mechanisms, and therapeutic applications.

Ligand	Type of NP	Size (nm)	Zeta Potential	Function/Target	Application	Ref.
IGF-II mAb	Liposomes	137.9–140.6	Positive	Targets IGF-II receptor to enhance BBB transport for gene delivery	Gene delivery for neurological disorders	[96]
scFv (TfR-targeted)	Liposomes	100	Positive	Targets TfR to improve nucleic acid delivery across BBB	Nucleic acid therapy; neuroinflammation treatment	[97]
OX26 mAb	SLNs	254 ± 17	Negative	Enhances BBB permeability and transcytosis efficiency for Alzheimer’s treatment	AD treatment	[98]
LB 509 mAb	SLNs	249 ± 1	Negative	Targets brain endothelial cells for drug delivery in AD	AD treatment	[98]
OX26 + DE2B4 mAbs	PLGA NPs	153–166	Negative	Dual targeting of TfR’s and Aβaggregates	ADAD, BBB permeability enhancement	[99]

**Table 4:** Estimated costs of various ligands commonly used in brain-targeted nanodelivery systems.

Ligand	Estimated Price per 100 mL of Solution (EUR)	Source
Tf	<25	[127]
Lf	<25	[128]
Protamine	<1	[129]
Insulin	<5	[130]
Chitosan	<1	[131]
ApoE3	>50 000	[132]
Ang-2	>5000 and <10,000	[133]
TAT	<35	[134]
B6 Peptide	<5	[135]
OX26 mAb	>50,000	[136]
LB 509 mAb	>10,000 and <15,000	[137]
DE2B4 mAbs	>5000 and <10,000	[138]
FasL	>15,000 and <20,000	[139]
Folate	<15	[140]
CDX	<5	[141]
CRT	<5	[141]
g7 Glycopeptide	>10,000 and <15,000	NA
dNP2 Peptide	<25	NA
IGF-II mAb	>10,000 and <15,000	NA

## Data Availability

Not applicable.

## Abbreviations

The following abbreviations are used in this manuscript:

Aβ	amyloid-beta
AD	Alzheimer’s disease
Ang-2	Angiopep-2
AMT	adsorptive-mediated transcytosis
ApoE3	Apolipoprotein E3
ApoE-SLNs	ApoE-functionalized solid lipid NPs
BBB	blood–brain barrier
BCECs	brain capillary endothelial cells
BDNF	brain-derived neurotrophic factor
BTE	brain tumor-related epilepsy
CAGR	compound annual growth rate
CBD	cannabidiol
CNS	central nervous system
CPP	cell-penetrating peptide
CRT	cyclic CRTIGPSVC
Cur	curcumin
DOX	doxorubicin
DXT	docetaxel
EMA	European Medicines Agency
FA	folic acid
FUS	focused ultrasound
Gb3	globotriaosylceramide
GMP	Good Manufacturing Practice
HA	hyaluronic acid
HSA	human serum albumin
LDL	low-density lipoprotein
Lf	lactoferrin
LfR	lactoferirn receptors
LPS	lipopolysaccharide
LRP-1	lipoprotein receptor-related protein 1
nAChR	nicotinic acetylcholine receptor
NGF	nerve growth factor
NLCs	nanostructured lipid carriers
NPs	nanoparticles
mAb	monoclonal antibody
MYR	myricetin
PBCECs	porcine brain capillary endothelial cells P
pBDNF	BDNF plasmid
PCB	polycarboxybetaine
PEG	polyethylene glycol
PLGA	polylactic-co-glycolic acid
PTX	paclitaxel
ROS	reactive oxygen species
RVG	rabies virus glycoprotein
SHK	shikonin
scFv	single-chain variable fragment
siRNA	interfering RNA
SLNs	solid lipid nanoparticles
STxB	shiga toxin B
TAT	transactivator of transcription peptide
Tf	transferrin
TfRs	transferrin receptors
TMC	N-trimethyl chitosan
TTF	tumor-treating fields
